# Combination of EZH2 and MEK inhibitors as an effective therapy for neurofibromatosis type 1-associated malignant peripheral nerve sheath tumors

**DOI:** 10.1093/noajnl/vdag221

**Published:** 2026-08-21

**Authors:** Sogo Oki, Yukitomo Ishi, Shigeru Yamaguchi, Manabu Natsumeda, Christine A Pratilas, Michiyuki Hakozaki, Hideki Tanizawa, Zen-ichi Tanei, Miki Fujimura

**Affiliations:** Department of Neurosurgery, Hokkaido University Graduate School of Medicine, Sapporo, Japan; Department of Neurosurgery, Hokkaido University Graduate School of Medicine, Sapporo, Japan; Department of Neurosurgery, Hokkaido University Graduate School of Medicine, Sapporo, Japan; Department of Neurosurgery, Brain Research Institute, University of Niigata, Niigata, Japan; Department of Oncology, The Sidney Kimmel Comprehensive Cancer Center, Johns Hopkins University School of Medicine, Baltimore, Maryland, USA; Department of Orthopaedic Surgery, Fukushima Medical University School of Medicine, Fukushima, Japan; Division of Genome Biology, Institute for Genetic Medicine, Hokkaido University, Sapporo, Japan; Department of Cancer Pathology, Hokkaido University Graduate School of Medicine, Sapporo, Japan; Department of Neurosurgery, Hokkaido University Graduate School of Medicine, Sapporo, Japan

**Keywords:** EZH2 inhibitor, MEK inhibitor, MPNST, NF1

## Abstract

**Background:**

Neurofibromatosis type 1 (NF1)-associated malignant peripheral nerve sheath tumors (MPNSTs) are aggressive sarcomas with poor outcomes and limited therapeutic options. Although mitogen-activated protein kinase kinase (MEK) inhibitors are active in benign plexiform neurofibromas, their efficacy in MPNST treatment is modest. Enhancer of zeste homolog 2 (EZH2) inhibitors are preclinically efficacious in MPNST treatment, but their mechanisms of action remain unclear. We evaluated the therapeutic potential and molecular mechanism of combined EZH2 and MEK inhibitors in NF1-associated MPNST.

**Methods:**

Five human NF1-associated MPNST cell lines were exposed to EZH2 and/or MEK inhibitors. Cell growth and apoptosis were quantified over time. Therapeutic efficacy was tested in a subcutaneous xenograft model. Proliferation and apoptosis in tumors were assessed using standard histologic markers, and intracellular localization of phosphorylated extracellular signal-regulated kinase (pERK) was examined using fluorescent immunohistochemistry.

**Results:**

Monotherapy with EZH2 or MEK inhibitors reduced proliferation and increased apoptosis across all MPNST lines. Combination therapy produced greater tumor cell growth suppression and marked increases in apoptosis. In vivo, the combination significantly delayed tumor progression compared with monotherapy, with concomitant reductions in proliferative indices and increases in apoptotic indices. EZH2 inhibitor limited nuclear pERK entry.

**Conclusions:**

Dual EZH2 and MEK inhibitors yield additive antitumor activity in NF1-associated MPNST. Although the molecular mechanism could not be elucidated, our findings suggest that EZH2 inhibitors exhibited a polycomb repressive complex 2-independent, noncanonical mechanism characterized by pERK nuclear translocation restriction, providing a strong rationale for clinical evaluation of this combination in NF1-associated MPNST.

Key Points

- Combination of EZH2 and MEK inhibitors produces additive antitumor effects.
- EZH2 inhibitors block pERK nuclear translocation via a PRC2-independent.
- Combination of EZH2 and MEK inhibitors significantly delays tumor progression in vivo.

Importance of the Study

NF1-associated malignant peripheral nerve sheath tumors (MPNST) lack effective therapies, as MEK inhibitors provide only transient benefit. This study reports that a combination of EZH2 and MEK inhibitors delivers additive antitumor effects across multiple NF1-associated MPNST cell lines in vitro and significantly delays tumor progression in vivo. Mechanistically, our findings suggest that EZH2 inhibitor restricts nuclear translocation of phosphorylated ERK via a PRC2-independent, noncanonical pathway; however, we were unable to elucidate the underlying molecular mechanism. These findings support clinical testing of combined EZH2 and MEK inhibitors in NF1-MPNST and highlight nuclear pERK localization as a therapeutic target.

Neurofibromatosis type 1 (NF1) is an autosomal dominant neurocutaneous tumor predisposition syndrome caused by germline pathogenic variants in the *neurofibromin 1* (*NF1*) tumor suppressor gene.^1^ It is characterized by the development of multiple peripheral nerve sheath tumors, most notably plexiform neurofibromas (PN), a subset of which may undergo malignant transformation into high-grade sarcomas called malignant peripheral nerve sheath tumors (MPNSTs).^2^ MPNST development is the major cause of decreased life expectancy in patients with NF1, occurring in 8%-13% of patients over their lifespan.^3^ Patients with NF1-associated MPNST have a 5-year survival rate of only 20%-50%,^4^ as these tumors have high rates of local recurrence and distant metastasis.^5^

Genomic profiling of MPNST cohorts revealed *NF1* mutations in 100% of NF1-associated MPNSTs and 82% of sporadic MPNSTs.^6^ Neurofibromin, the product of *NF1*, negatively regulates RAS activity. Lack of functional neurofibromin in patients with NF1 leads to dysregulated RAS expression and tumorigenesis with acceleration of the mitogen-activated protein kinase (MAPK) cascade.^7^ This is an intracellular signaling pathway comprising sequential phosphorylation and activation of Ras, Raf, MAPK kinase (MEK), and extracellular signal-regulated kinase (ERK), which regulate a large number of cellular processes including proliferation and differentiation.^8-10^ Selumetinib, an oral selective MEK inhibitor, has shown impressive clinical activity in children and adolescents with PN with a durable and significant decrease in tumor volume.^11-14^ However, in MPNST, MEK inhibitors do not completely suppress tumor growth, demonstrating only moderate and transient preclinical effects.^15-17^ Indeed, long-term administration of the MEK inhibitor trametinib to genetically modified mouse models of MPNST was reported to induce rapid kinase reprogramming through extensive activation of receptor tyrosine kinases (RTKs) and intracellular signaling, thereby promoting treatment resistance.^18^^,^^19^ Thus, MEK inhibitors may be more effective when used in combination with other therapies.

Enhancer of zeste homolog 2 (EZH2), a histone-lysine N-methyltransferase and a polycomb group protein, is a critical component of polycomb repressive complex 2 (PRC2).^20^ PRC2 plays an essential role in the epigenetic maintenance of the repressive trimethylated histone H3K27 (H3K27me3) chromatin mark.^21^ However, loss-of-function somatic alterations in different components of PRC2, *EED*, or *SUZ12* have been detected in most MPNST cases,^22-24^ and the role of EZH2 in MPNST pathogenesis is poorly understood.^25^ In contrast, reports have demonstrated the therapeutic efficacy of EZH2 inhibitors in MPNST.^25^^,^^26^ The proposed pharmacological mechanism involves EZH2/KPNB1 signaling downregulation, thereby hindering the nuclear translocation of phosphorylated ERK (pERK).^26^

In this study, we present in vitro and in vivo data targeting pERK nuclear translocation by EZH2 inhibitors in combination with inhibition of the MAPK cascade as a treatment strategy for patients with MPNST. Simultaneous inhibition of EZH2 and MEK exhibits cytotoxic activity in vitro against NF1-associated MPNST cells. This combination regimen was also active in vivo (eg, delayed tumor progression) in an NF1-associated MPNST xenograft mouse model. Together, these findings indicate that this novel regimen rationally combining EZH2 and MEK inhibitors might represent a promising option for the treatment of NF1-associated MPNST.

## Methods

### MPNST Cell Lines

We used five human NF1-associated MPNST cell lines in this study: sNF96.2, sNF02.2, JH-2-103, JH-2-002, and FMS-1. sNF96.2 and sNF02.2 were purchased from the American Type Culture Collection. JH-2-103 and JH-2-002 were kindly provided by Dr. Christine Pratilas through the Johns Hopkins NF1 biospecimen repository.^27-29^ FMS-1 was obtained from Dr. Michiyuki Hakozaki (Department of Orthopedic Surgery, Fukushima Medical University, Japan).^30^ The primary human Schwann cell line HMP303 was purchased from Neuromics, Inc. (Neuromics Inc., Edina, MN, USA). The MPNST cell lines were maintained in Dulbecco’s modified Eagle medium supplemented with 10% fetal bovine serum and 1% antibiotics (penicillin and streptomycin) at 37 °C in a humidified 5% CO_2_ atmosphere. HMP303, the cell line of normal human Schwann cells, was maintained in Schwann Growth Media (Neuromics Inc.) in a flask or plate coated with AlphaBiocoat Solution (Neuromics Inc.).

### Cell Proliferation and Apoptosis Assays

EZH2 inhibitors GSK126 (Selleck Chemicals LLC, Houston, TX, USA) and tazemetostat (Focus Biomolecules, PA, USA) and the MEK inhibitor selumetinib (Selleck Chemicals LLC) were dissolved in dimethyl sulfoxide (DMSO) to yield the appropriate stock concentrations and then frozen at −80 °C until further use.^31^ Cell proliferation was determined using the CellTiter 96 AQueous One Solution Cell Proliferation Assay (Promega, Madison, WI, USA) according to the manufacturer’s recommendations. After treatment of MPNST cells with 100 μL of medium in 96-well plates, 20 μL of MTS solution was added to each well and incubated at 37 °C for 2 hours. The 96-well plates were then placed in a Sunrise microplate absorbance reader (TECAN, Männedorf, Switzerland), and the absorbance was measured at 490 nm.

The effect of GSK126 and selumetinib on MPNST cell proliferation was determined in 96-well plates using the MTS assay. First, for the determination of cell proliferation effects and safety of inhibitor treatments, MPNST cells and normal Schwann cells were seeded in 96-well plates at 1,000 cells per well. After the cells were allowed to adhere to the substratum overnight, they were cultured for 72 h in the presence of different concentrations of selumetinib and GSK126 (1, 5, 10, 15, 20, and 30 μM). The 50% growth inhibition (IC_50_) values were calculated using nonlinear least-squares curve-fitting.

Next, to compare the effects of the inhibitors, the five MPNST cell lines were seeded in 96-well plates at 1,000 cells per well. After the cells were allowed to adhere to the substratum overnight, they were cultured in the presence of either vehicle control (DMSO) or the IC_50_ of selumetinib and/or GSK126. Cell proliferation was analyzed on days 1, 3, 5, 7, and 10 using the MTS assay. We performed the same experiment using tazemetostat instead of GSK126 as the EZH2 inhibitor.

For the apoptosis assay, the MPNST cells were seeded and cultured in T25 flasks in the presence of selumetinib and/or GSK126 for 5-7 days. Apoptosis was assessed using the Annexin V-FITC kit (Medical & Biological Laboratories Co., Ltd., Tokyo, Japan) using BD FACSVerse (BD Biosciences, Franklin Lakes, NJ, USA).

### RNA-Seq and Analysis

sNF96.2 cells were cultured as described above and treated with either a control vehicle (0.05% DMSO) or GSK126 and/or selumetinib for 5 days. The GSK126 and selumetinib concentrations were each set to their respective IC_50_ values. RNA was extracted from three biological replicates per condition using the Allprep DNA/RNA Mini Kit (QIAGEN, Hilden, Germany) according to the manufacturer’s instructions. RNA sequencing data were obtained through outsourcing to BGI (BGI Genomics, Guangdong, China). Sequencing libraries were generated using the Optimal Dual-mode mRNA Library Prep Kit, which incorporates a poly(A) selection step. The purified RNA was sequenced (150bp paired-end directional reads; more than 22 M reads per sample) on DNBSEQ-G400. Read counts were normalized using DESeq2 size factors. The differentially expressed genes (DEGs) were analyzed using DESeq2 (version 1.38.3) with R, by applying thresholds of |log2FoldChange (log2FC)| ≥1 and a false discovery rate (FDR) < 0.05. *P*-values were adjusted using the Benjamini-Hochberg method.^32^ Principal component analysis (PCA) was performed on variance-stabilized counts obtained using the vst function in DESeq2. Gene ontology (GO) enrichment analysis was performed using Metascape (http://metascape.org). The differentially expressed genes (up- and downregulated) were submitted and analyzed in the “Express Analysis” mode. The differentially expressed genes were ranked based on log2FC, and the top 20 down- and upregulated pathways in response to the treatment were plotted.

To investigate expression changes in the MAPK cascade, we retrieved 267 genes associated with the KEGG MAPK signaling pathway from the Molecular Signatures Database (https://www.gsea-msigdb.org/gsea/msigdb). We identified 62 DEGs that met the significance criteria |log2FC| ≥1 and an FDR <0.05 in at least one pairwise comparison between conditions from that set. The expression profiles of these 62 genes were visualized using a heatmap to compare the changes across conditions.

### Western Blot Analyses

The MPNST cell lines were seeded and cultured in growth media and treated for 3 days with vehicle control (dimethylsulfoxide, DMSO) or the IC_50_ of selumetinib and/or GSK126. Protein lysates were collected from the cultures after 3 days using a RIPA Lysis Buffer System (Santa Cruz Biotechnology, USA). Histone protein lysates were collected from cultures in growth media using the Histone Extraction Kit (#ab113476; Abcam, UK). Protein concentration was quantified by Pierce BCA Protein Assay Kits (Thermo Fisher Scientific, USA). Equal amounts of total protein (2 μg/lane) were subjected to sodium dodecyl sulfate-polyacrylamide gel electrophoresis and subsequently transferred to a polyvinylidene fluoride membrane by electroblotting. The remaining protein binding sites of the membrane were blocked using phosphate-buffered saline, containing 0.05% Tween 20 and Amersham ECL Prime blocking agent (2%). Primary antibody incubations were performed in the same buffer with the following primary antibodies: rabbit monoclonal antibodies against pMAPK P44/42 (#4370; Cell Signaling Technology, Inc., Danvers, MA, USA), mouse monoclonal antibodies against β-Actin (Santa Cruz Biotechnology, USA), mouse monoclonal anti-Histone H3K27 (trimethyl H3K27) antibodies (#ab6002; Abcam, UK), mouse monoclonal antibodies against Histone H3 (#3638; Cell Signaling Technology, Inc., Danvers, MA, USA), and mouse monoclonal anti-KPNB1 antibodies (#ab2811; Abcam, UK). Horseradish peroxidase-conjugated donkey-anti-rabbit (#NA934VS; Cytiva, USA) and sheep-anti-mouse (#NA931VS; Cytiva, USA) were used as secondary antibodies. Clarity Western ECL Substrate (Bio-Rad, USA) was used for visualizing the signal under a ChemiDoc Imaging System (Bio-Rad, USA).

### Xenograft Studies

All the animal procedures were approved by the Animal Studies Ethics Committee of the Hokkaido University Graduate School of Medicine (approval number: 24-0063). All the experimental procedures were conducted in accordance with the Institutional Guidelines for Animal Experimentation and the Guidelines for Proper Conduct of Animal Experiments by the Science Council of Japan. We purchased 6-8-week-old female severe combined immunodeficiency (SCID) mice (scid/scid genotype, C.B17/Icr-scidJcl background) from CLEA Japan. In these experiments, 2 × 10^7^ JH-2-103 cells were transplanted into the subcutis of the right flank of these mice. When the tumor size entered the growth phase (18 days after cell inoculation), the mice were randomly divided into four groups: (i) control (DMSO, *n* = 5), (ii) GSK126 monotherapy (100 mg/kg for 4 weeks, *n* = 5) (iii) selumetinib monotherapy (5 mg/kg, two times daily, *n* = 5), and (iv) GSK 126 and selumetinib combination therapy (*n* = 5). GSK126 was administered via intraperitoneal injection once a day for 35 days (5 days a week for 5 weeks) from 20 days after tumor cell implantation. Selumetinib was administered twice a day orally for 35 days (5 days a week for 5 weeks) starting 20 days after tumor cell implantation. The GSK126 and selumetinib doses were chosen based on previous studies that reported doses that are both safe and effective for mice.^33^^,^^34^

Tumor size and weight were measured twice a week. The tumor volume was calculated using the formula L × W^2^ × 0.5, where L represents the longest diameter and W, the width, as previously described in the literature.^35^ At each designated point, the mean tumor volumes among the four groups were compared. Each mouse was euthanized once its tumor reached 1000 mm^3^. Following euthanasia, the tumors were excised and fixed in 10% formalin. Tumors were prepared for histopathological evaluation. Indications of toxicity were evaluated by weight loss (>20% of initial weight) and inability to move, as described previously.^33^

### Immunohistochemistry

Tumor tissues were collected from the mice at completion of the last treatment (*n* = 1 for each treatment). Formaldehyde-fixed tissues were paraffin-embedded and sectioned (4 μm) for hematoxylin and eosin and anti-Ki-67 antibody (clone MIB-1, #M7240; DAKO, Carpinteria, CA, USA) staining. To assay apoptotic responses to treatment, TUNEL staining was performed using the ApopTag Fluorescein In Situ Apoptosis Detection Kit (Merck KGaA, Darmstadt, Germany) according to the manufacturer’s protocol for paraffin-embedded tissues. For the evaluation of pERK localization, fluorescent immunostaining was performed. Primary antibody incubations were carried out in the same buffer using the following primary antibodies: rabbit monoclonal antibodies against pMAPK P44/42 (#4370; Cell Signaling Technology, Inc., Danvers, MA, USA). Horseradish peroxidase conjugated goat-anti-rabbit (Alexa Fluor 488, #A11008; Thermo Fisher Scientific Inc., Waltham, MA, USA) was used as a secondary antibody. All images were acquired and analyzed using an all-in-one fluorescence microscope (BZ-X800; Keyence, Osaka, Japan).

### Immunocytochemistry

Cells (200,000) were seeded into 2-well chamber slides and incubated with the IC_50_ value of GSK126 for 72 h. Four percent paraformaldehyde was used for fixing tumor cells on slides. Subsequent procedures, including primary antibody reaction, secondary antibody reaction, and imaging, were performed as the same as that of immunohistochemistry. Nuclear-dominant pERK-positive cells were quantified using immunofluorescence analysis. For each cell, the nuclear-to-cytoplasmic pERK fluorescence ratio was calculated as the background-corrected mean nuclear pERK fluorescence intensity divided by the background-corrected mean cytoplasmic pERK fluorescence intensity. Nuclei were detected with DAPI. Cells with a nuclear-to-cytoplasmic pERK ratio greater than 1.5 were defined as nuclear-dominant cells and were analyzed using BZ-X Analyzer (Keyence, Osaka, Japan). The percentage of nuclear-dominant cells was calculated in the MPNST cells treated with DMSO and GSK126.

### Statistical Analysis

Survival plots were generated and analyzed using the Kaplan-Meier method and Prism 10 (GraphPad), and *P*-values were calculated using the log-rank test. Welch’s unpaired *t*-test was used for the immunofluorescence analysis. For all other analyses, one-way analysis of variance (ANOVA) and Tukey’s multiple comparisons were used to determine the significance of differences between treatment groups. A *P*-value <.05 was considered statistically significant.

## Results

### Targeted Inhibition of EZH2 and MEK Activity Reduces Cell Proliferation and Induces Apoptosis of NF1-Associated MPNST Cells

**Figure 1.**
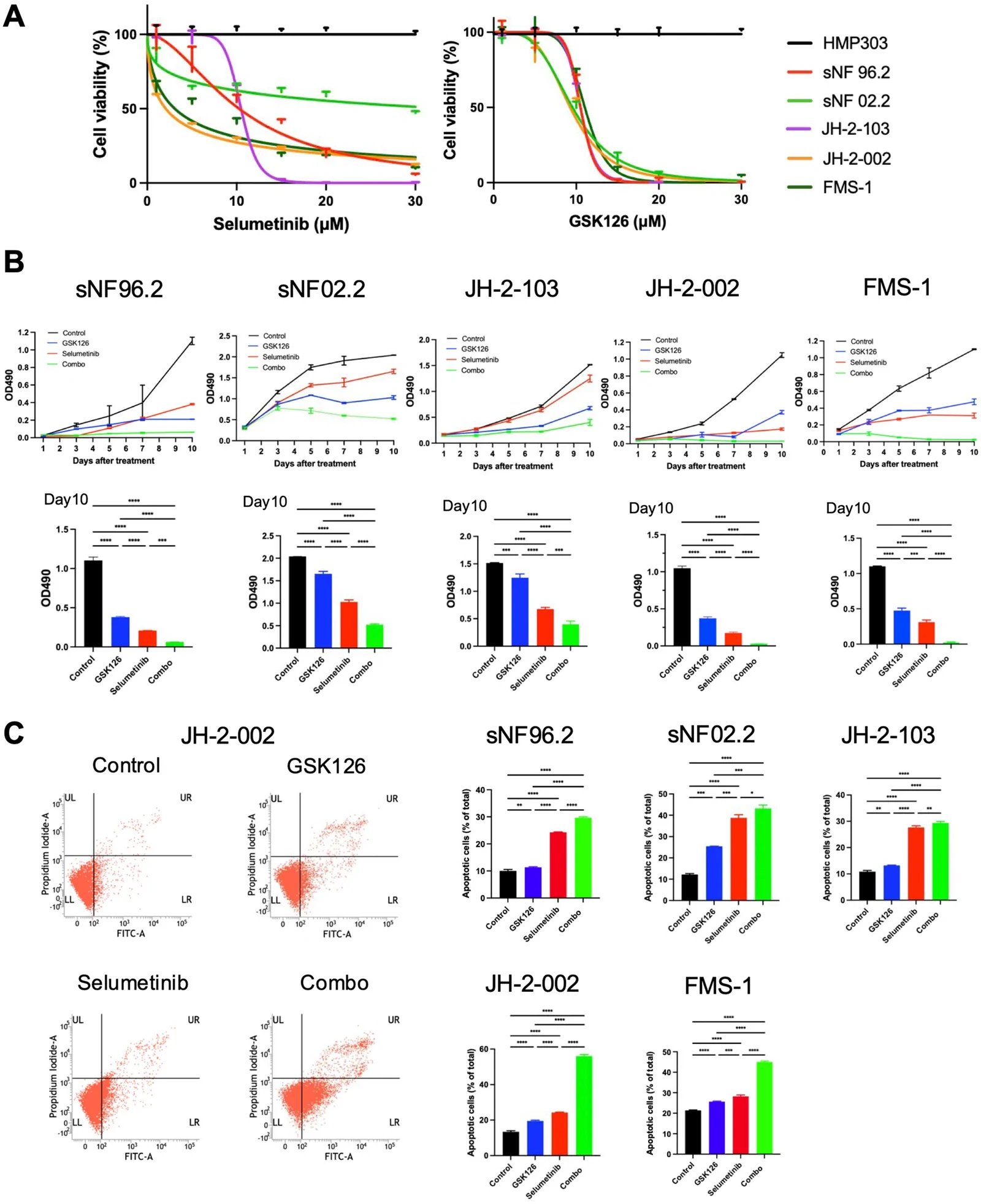
Inhibition of EZH2 and MEK activity reduced proliferation and induced apoptosis in NF1-associated MPNST cells. (A) Dose-response curve of sNF96.2, sNF02.2, JH-2-103, JH-2-002, FMS-1, and HMP303 cells treated for 3 days with the indicated concentrations of GSK126 and selumetinib. (B) Cell growth (upper) plot showing antiproliferative effects of GSK126 and selumetinib in NF1-associated MPNST cells. The plot represents the normalized metabolic activity by absorbance measured on days 1, 3, 5, 7, and 10. Values shown are the average (mean SD) from duplicate or triplicate samples for each condition. The bar graph (lower) represents the normalized metabolic activity on day 10. One-way ANOVA values for comparisons between each condition. sNF96.2: *P *< .0001; ****, *P *< .0001 for control versus GSK126; ****, *P *< .0001 for control versus selumetinib; ****, *P *< .0001 for control versus combo; ****, *P *< .0001 for GSK126 versus selumetinib; ****, *P *< .0001 for GSK126 versus combo; ***, *P *= .0002 for selumetinib versus combo. sNF02.2: *P *< .0001; ****, *P *< .0001 for control versus GSK126; ****, *P *< .0001 for control versus selumetinib; ****, *P *< .0001 for control versus combo; ****, *P *< .0001 for GSK126 versus selumetinib; ****, *P *< .0001 for GSK126 versus combo; ****, *P *< .0001 for selumetinib versus combo. JH-2-103: *P *< .0001; ***, *P *= .0006 for control versus GSK126; ****, *P *< .0001 for control versus selumetinib; ****, *P *< .0001 for control versus combo; ****, *P *< .0001 for GSK126 versus selumetinib; ****, *P *< .0001 for GSK126 versus combo; ***, *P *= .0005 for selumetinib versus combo. JH-2-002: *P *< .0001; ****, *P *< .0001 for control versus GSK126; ****, *P *< .0001 for control versus selumetinib; ****, *P *< .0001 for control versus combo; ****, *P *< .0001 for GSK126 versus selumetinib; ****, *P *< .0001 for GSK126 versus combo; ****, *P *< .0001 for selumetinib versus combo. FMS-1: *P *< .0001; ****, *P *< .0001 for control versus GSK126; ****, *P *< .0001 for control versus selumetinib; ****, *P *< .0001 for control versus combo; ***, *P *= .0001 for GSK126 versus selumetinib; ****, *P *< .0001 for GSK126 versus combo; ****, *P *< .0001 for selumetinib versus combo. (C) NF1-associated MPNST cells were treated with GSK126 or selumetinib or both, after which flow cytometric analysis was performed to determine the percentage of Annexin V^+^ apoptotic cells. Left, the representative results (sNF96.2) are shown. Right, the data for at least three independent experiments are presented as bar graphs. One-way ANOVA for comparisons between treatments. sNF96.2: *P *< .0001; **, *P *= .0047 for control versus GSK126; ****, *P *< .0001 for control versus selumetinib; ****, *P *< .0001 for control versus combo; ****, *P *< .0001 for GSK126 versus selumetinib; ****, *P *< .0001 for GSK126 versus combo; ****, *P *< .0001 for selumetinib versus combo. sNF02.2: *P *< .0001; ***, *P *= .0009 for control versus GSK126; ****, *P *< .0001 for control versus selumetinib; ****, *P *< .0001 for control versus combo; ***, *P *= .0008 for GSK126 versus selumetinib; ***, *P *= .0003 for GSK126 versus combo; *, *P *= .0493 for selumetinib versus combo. JH-2-103: *P *< .0001; ***, *P *= .0001 for control versus GSK126; ****, *P *< .0001 for control versus selumetinib; ****, *P *< .0001 for control versus combo; ***, *P *= .0001 for GSK126 versus selumetinib; ****, *P *< .0001 for GSK126 versus combo; ****, *P *< .0001 for selumetinib versus combo. JH-2-002: *P *< .0001; ****, *P *< .0001 for control versus GSK126; ****, *P *< .0001 for control versus selumetinib; ****, *P *< .0001 for control versus combo; ****, *P *< .0001 for GSK126 versus selumetinib; ****, *P *< .0001 for GSK126 versus combo; ****, *P *< .0001 for selumetinib versus combo. FMS-1: *P *< .0001; ****, *P *< .0001 for control versus GSK126; ****, *P *< .0001 for control versus selumetinib; ****, *P *< .0001 for control versus combo; ***, *P *= .0004 for GSK126 versus selumetinib; ****, *P *< .0001 for GSK126 versus combo; ****, *P *< .0001 for selumetinib versus combo.

We hypothesized that EZH2 and MEK inhibitors suppress the growth of NF1-associated MPNST by blocking distinct nodes of the MAPK signaling pathway. At the drug concentrations employed in this study, no cytotoxic effects were observed on normal Schwann cells (Figure 1A). We first treated NF1-associated MPNST cell lines with GSK126 and selumetinib, a MEK inhibitor, and assessed their effects on cell proliferation and apoptosis. Monotherapy with either GSK126 or selumetinib suppressed the growth of all five NF1-associated MPNST cell lines in a time-dependent manner (Figure 1A and B). Although the extent of the MAPK signaling varied between cell lines (Figure S1), antitumor activity by selumetinib was observed in all cell lines (Figure 1A). Similarly, each single-agent treatment significantly induced apoptosis across all cell lines. Combination therapy with GSK126 and selumetinib further suppressed proliferative activity and enhanced apoptosis in MPNST cells compared with monotherapy (Figure 1B and C; Figure S2). Moreover, another clinically available EZH2 inhibitor, tazemetostat, inhibited the metabolic activity of MPNST cell lines in a dose-dependent manner, and its combination with selumetinib led to an even greater suppression of cellular metabolic activity (Figure S3).

### Effects of EZH2 and MEK Inhibition on Gene Expression in NF1-Associated MPNST Cells

**Figure 2.**
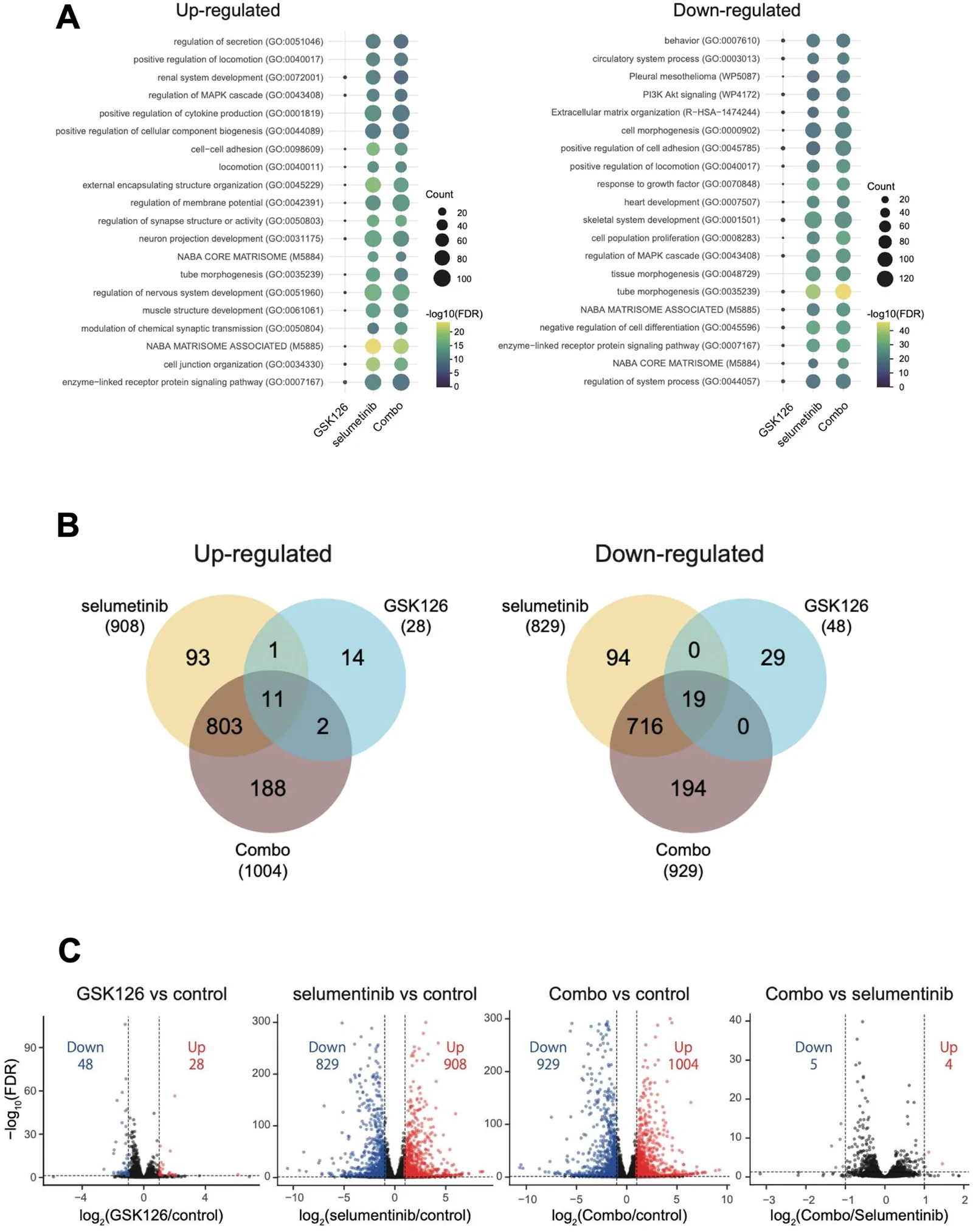
RNA sequencing revealed genome-wide gene expression profiles of sNF96.2 cells treated with GSK126 and selumetinib alone or in combination. (A) GO enrichment pathway analysis in sNF96.2 cells. (B) Venn diagram showing the number of genes that are differentially expressed (log2FC ≥ 1, FDR < 0.05) and their relationship after treatment with GSK126 and selumetinib or in combination, compared to untreated control. (C) Volcano plot of differentially expressed genes (DEGs). DEGs were identified using the DESeq2 R package.

To elucidate the impact of targeted inhibition of EZH2 and/or MEK activity on gene expression, we performed comprehensive transcriptomic profiling by RNA-seq using NF1-associated MPNST cells treated with DMSO, GSK126, and/or selumetinib. PCA revealed that the three replicates under each condition clustered closely together, confirming good experimental reproducibility (Figure S4A). However, GSK126 treatment alone induced only minimal transcriptional changes compared with DMSO (Figure 2A and B; Figure S4B and C).

Gene ontology (GO) pathway enrichment analysis revealed that GSK126 monotherapy induced only minimal alterations in gene expression, whereas selumetinib monotherapy and the combination therapy resulted in substantial alterations (Figure 2A). Moreover, the analysis of the dataset for the significantly downregulated or upregulated transcripts (log2FC ≥1, FDR <0.05) revealed gene expression profiles (GEPs) for GSK126 (blue) and selumetinib (yellow) or in combination (brown) (Figure 2B; Table S1). GSK126 monotherapy induced minimal changes in gene expression. In both selumetinib monotherapy and combination therapy, most gene expression changes were shared (Figure 2C). Analysis of the 62 MAPK pathway-related genes showed that GSK126 monotherapy induced a slight change in gene expression, whereas selumetinib monotherapy and the combination treatment caused marked transcriptional alterations, with largely overlapping transcriptional profiles (Figure S4C). Western blot analysis of pERK, used as a surrogate marker of MAPK pathway activation, showed a small change in pERK levels after GSK126 monotherapy, whereas pERK levels decreased following selumetinib monotherapy and the combination treatment (Figure S5). Since the majority of MPNST harbored genetic alterations in the genes in PRC components and showed subsequent loss of H3K27me3,^22^ we assessed histone H3K27 trimethylation (H3K27me3) status in each cell line and found that all cell lines showed loss of H3K27me3 except one cell line (sNF02.2) with PRC2 loss (Table 1; Figure S6). To evaluate the downregulation of EZH2/KPNB1 signaling, we examined KPNB1 expression following GSK126 treatment; however, reduced KPNB1 expression was observed only in one cell line (Figure S7).

**Table 1.** Characteristics of MPNST cell lines

Cell line	Origin	PRC2 status	SUZ12 status	H3K27me3 status
sNF96.2	NF1	Loss^36^	Mutant^36^	Loss
sNF02.2	NF1	Retained^37^	Wildtype^36^	Retained
JH-2-103	NF1	Loss^38^	Mutant^a^	Loss
JH-2-002	NF1	Loss^38^	Mutant^a^	Loss
FMS-1	NF1	NA	Unknown	Loss

a From Johns Hopkins NGS-based profiling.

### Antitumor Effects of EZH2 and MEK Inhibitors in Subcutaneous NF-Associated MPNST Xenograft

**Figure 3.**
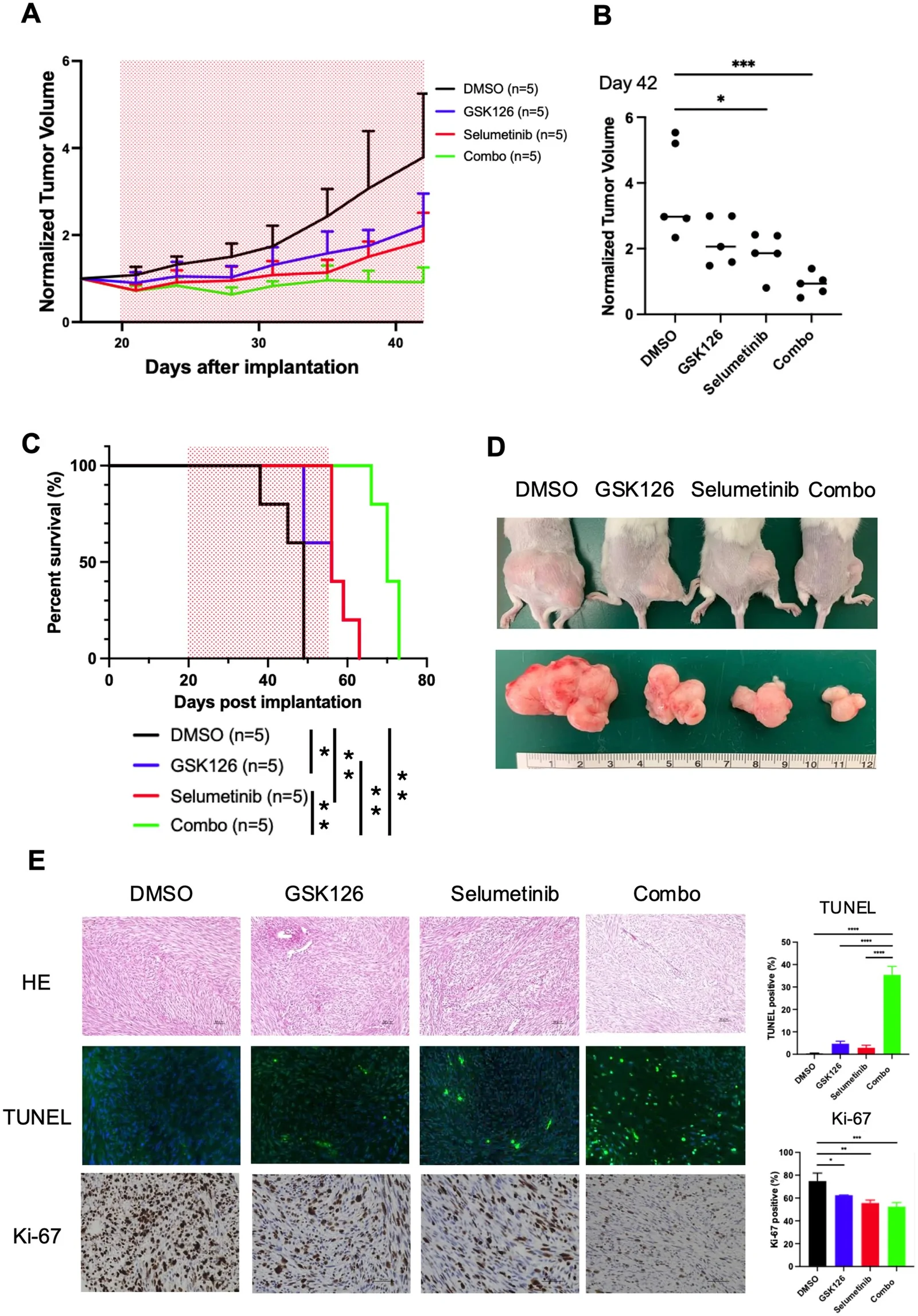
Treatment with EZH2 and MEK inhibitors showed antitumor activity in xenograft models of NF1-associated MPNST. Mice with JH-2-103 subcutaneous tumors were either treated with vehicle (DMSO, *n* = 5), GSK126 (100 mg/kg for 35 consecutive days, *n* = 5), selumetinib (50 mg/kg, twice a day for 35 consecutive days, *n* = 5), or combination (same dose of monotherapy for 35 consecutive days, *n* = 5). (A) Normalized tumor volume over the course of postimplantation treatment with DMSO, GSK126, selumetinib, or combination. (B) Dot plot representation of normalized tumor volume in mice at day 42 post-tumor cell injection. Horizontal bars indicate the mean value for each treatment group. One-way ANOVA for comparisons between treatments: *P *= .0011; DMSO versus selumetinib; *, *P *= .0174; DMSO versus combo; **, *P *= .0006. (C) Mice were sacrificed after drug treatment for 35 days, and then the images of the mice and tumors were captured. (D) Corresponding survival plots for each experiment. Statistical analysis was performed using a log-rank test: DMSO versus GSK126; *, *P *= .0291; DMSO versus selumetinib; **, *P *= .0034; DMSO versus combo; **, *P *= .0034; GSK126 versus combo; **, *P *= .0017; selumetinib versus combo; **, *P *= .0015. (E) Left, images of representative hematoxylin and eosin, TUNEL, and Ki-67 staining of subcutaneous tumors from mice euthanized at the end of treatment. Right, mean and SD values representing the average number of positive cells in four high-powered fields in each tumor. One-way ANOVA for comparisons between treatments. TUNEL: *P *< .0001; ****, *P *< .0001 for DMSO versus combo; ****, *P *< .0001 for GSK126 versus combo; ****, *P *< .0001 for selumetinib versus combo. Ki-67: *P *= .0008; *, *P *= .0264 for DMSO versus GSK126; **, *P *= .021 for DMSO versus selumetinib; ***, *P *= .0008 for DMSO versus combo.

Based on the observed effects of GSK126 and selumetinib on the growth of NF1-associated MPNST cells, we hypothesized that targeting a distinct MAPK signaling pathway was an effective therapeutic approach and that inhibition of EZH2 and MEK activities suppressed tumor proliferation in mice bearing NF1-associated MPNST xenografts. To test this hypothesis, we treated NF1-associated MPNST xenografts (JH-2-103) with GSK126 and selumetinib, alone or in combination. Before conducting the efficacy study of GSK126 and selumetinib, we evaluated their clinical toxicities based on weight loss (>20% of initial weight) and inability to move. We observed no toxicity in mice treated with 100 mg/kg GSK126 once daily and/or 50 mg/kg selumetinib twice daily (Figure S8). To evaluate the in vivo antitumor activity, mice with subcutaneous NF1-associated xenografts were treated with 100 mg/kg once daily GSK126 and/or 50 mg/kg twice daily selumetinib, alone or in combination, for 25 days (5 days a week for 5 weeks). Both GSK126 and selumetinib monotherapies reduced tumor growth in NF1-associated MPNST xenograft models, as evidenced by decreased tumor volume and improved survival in treated mice (Figure 3A-D). The combination treatment exhibited significantly reduced tumor size compared with the vehicle group (*P *= .0006), although the differences versus either monotherapy were not statistically significant at day 42 post-tumor cell injection (Figure 3B). We found that combination treatment of GSK126 and selumetinib significantly improved survival rate, outperforming either monotherapy (Figure 3C). This study included mice that were euthanized at the end of treatment to obtain subcutaneous tumor samples for analyzing tumor cell proliferation (Ki-67) and apoptosis (TUNEL; Figure 3D). Analysis of intratumor Ki-67 staining showed that although combination treatment did not differ significantly from either monotherapy group, it significantly reduced the tumor cell proliferation relative to the vehicle treatment group (*P* = .0008; Figure 3E). TUNEL staining results showed the highest proportion of positive cells in tumors from mice receiving combination therapy of GSK126 and selumetinib relative to either monotherapy (*P* < .0001 relative to GSK126 alone, *P* < .0001 relative to selumetinib alone in Figure 3E).

### Nuclear Entry of pERK Is Limited by EZH2 Inhibitor

**Figure 4.**
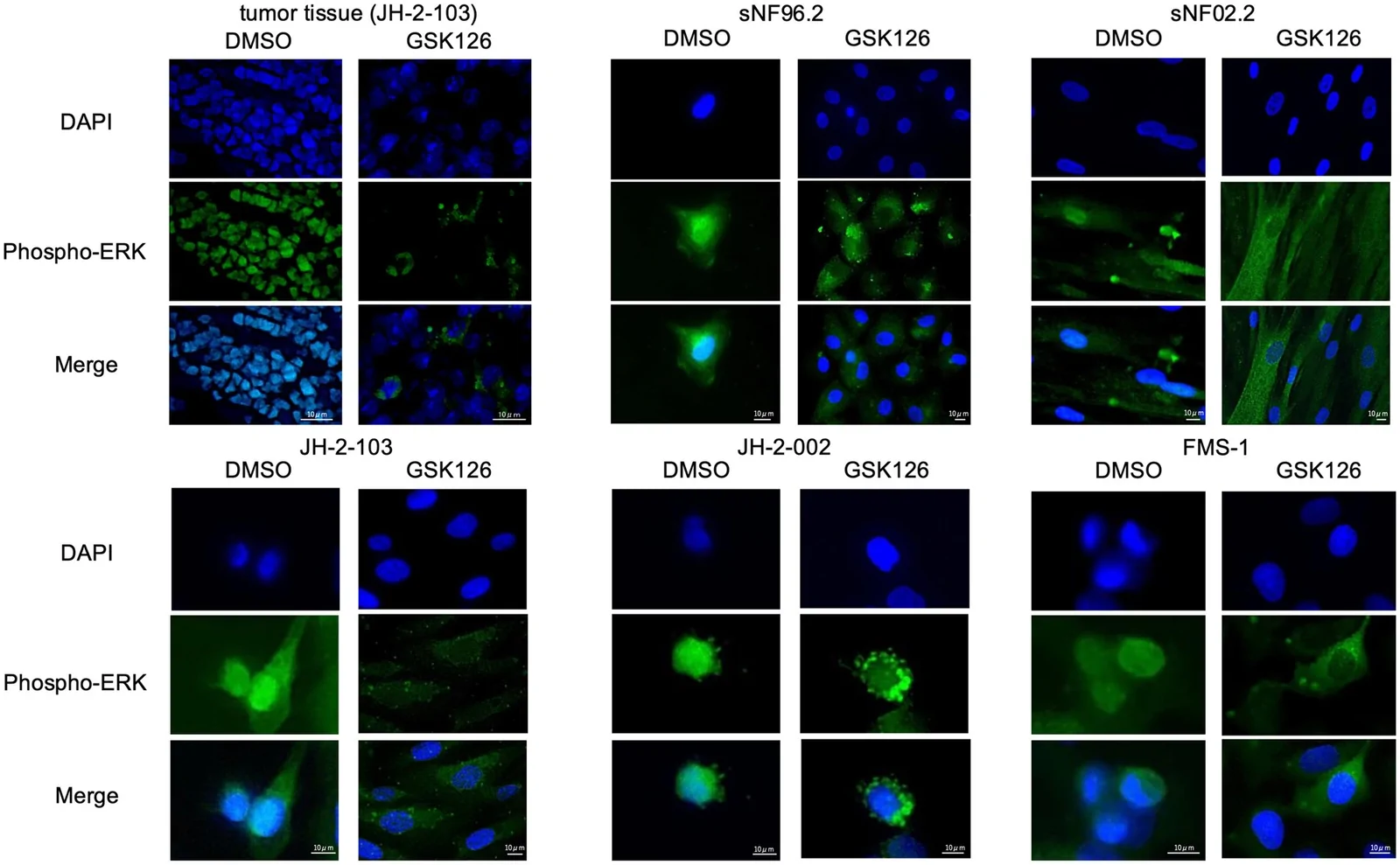
EZH2 inhibition suppresses the nuclear localization of pERK in vivo and in vitro. In the DMSO group, strong nuclear localization of pERK was observed, whereas in the GSK126 group, pERK remained confined to the cytoplasm.

Using subcutaneous tumor samples and cultured cell lines, we evaluated the localization of pERK by immunofluorescence staining (Figure 4). In the vehicle treatment group, pERK localized predominantly to the nuclei of cells. However, in the GSK126 treatment group, pERK remained principally cytoplasmic. Immunofluorescence analysis showed that the proportion of nuclear-dominant pERK-positive cells was significantly decreased in the GSK126 treatment group compared with the vehicle treatment group (Figure S9).

## Discussion

MPNSTs are aggressive soft-tissue sarcomas that represent an important clinical challenge, particularly given their strong tendency to relapse and metastasize and their relatively poor response to conventional therapies.^39^ As a novel treatment, molecularly targeted drugs are highly anticipated. While limitations in the therapeutic efficacy of MEK inhibitors as monotherapy for MPNST have been reported, there are also increasing reports on the combination effects with other molecularly targeted drugs (such as inhibitors of mTOR, AKT, CDK4/6, SHP2, BMP, TYK2, MET, and PDGFRβ inhibitors).^17^^,^^19^^,^^40-45^ However, none of these have been established as standard treatments.

The therapeutic efficacy of EZH2 inhibitors against MPNST was reported in preclinical studies.^25^^,^^26^ Clinical application is also anticipated, with a Phase II clinical trial currently underway (ClinicalTrials.gov identifier NCT04917042). EZH2 is the catalytic subunit of PRC2, which is a highly conserved histone methyltransferase that targets lysine-27 of histone H3.^46^ PRC2 functions as a transcription repressor and plays a crucial role in regulating gene expression and suppression during numerous physiological and developmental processes. These processes include stem cell maintenance, cellular senescence, cell differentiation, and cell fate determination.^47^ Therefore, EZH2 histone methyltransferase has emerged as a key target in potential epigenetic strategies.^31^^,^^46^ However, 60%-70% of MPNST cases harbor inactivating somatic mutations in *SUZ12* or *EED*—both core components of the PRC2 complex alongside EZH2—resulting in the loss of PRC2 activity.^22^^,^^48^ In MPNST, when PRC2 exhibits loss of function, EZH2 inhibitors are unlikely to alter gene expression through epigenetic modification. Table 1 summarizes the PRC2, SUZ12, and H3K27me3 status of the MPNST cell lines used in the present study. PRC2 and SUZ12 statuses were evaluated based on genetic alterations.^36-38^ Evaluating H3K27me3 status, all but one cell line showed loss of H3K27me3 and only sNF02.2 with wild-type PRC2 showed retained H3K27me3. Indeed, pathway analysis of RNA sequencing data from PRC2-deficient MPNST cells (sNF96.2) treated with an EZH2 inhibitor demonstrated only limited changes across pathways. These results suggest that EZH2 has noncanonical functions beyond its role as a PRC2 component.

Zhang et al reported the efficacy of EZH2 inhibitors against MPNST.^25^ They elucidated that the EZH2-miR-30d-KPNB1 pathway is involved in this mechanism. Suppressing EZH2 increases miRNA30d expression and decreases KPNB1 expression, thereby reducing the nuclear translocation of pERK and inducing apoptosis.^26^ Inhibition of pERK nuclear translocation has demonstrated antitumor efficacy in other cancers.^49^^,^^50^ Intriguingly, because cytoplasmic pERK is preserved, this approach is thought to minimally perturb pERK-dependent negative feedback loops.^49^ In our study, EZH2 inhibition suppressed NF1-associated MPNST tumor growth; however, pathway analysis of RNA-sequencing data revealed minimal perturbations. This finding is consistent with those of previous reports.^25^^,^^42^ In our present study, despite the coexistence of both wild-type and loss of PRC2 status,^37^^,^^38^ we observed consistent antitumor effects of EZH2 inhibitors, as well as additive efficacy when combined with MEK inhibitors, across all the tested cell lines. This strongly indicates that in NF1-associated MPNST, EZH2 inhibitors exert their activity through a PRC2-independent mechanism, such as inhibition of pERK nuclear translocation. Combining MEK inhibition with pERK nuclear translocation blockade was reported to produce synergistic antitumor activity in BRAF-, NRAS-, and NF1-mutant melanomas.^50^ This regimen nearly abolishes pERK nuclear translocation while preserving cytoplasmic pERK, thereby maintaining MAPK pathway-dependent negative feedback loops and yielding robust antitumor effect. Notably, inhibition of pERK nuclear translocation may also suppress or delay the emergence of resistance to MEK inhibitors. Michailovici et al demonstrated the efficacy of inhibition of pERK nuclear translocation in BRAF-mutant melanoma with acquired MEK-inhibitor resistance.^49^ Consistent with these reports, our experiments in NF1-associated MPNST demonstrated that combining an MEK inhibitor with EZH2 inhibitor-mediated blockade of pERK nuclear translocation elicited additive antitumor activity.

The present study has some limitations. First, we did not assess whether *EZH2* knockdown suppresses NF1-associated MPNST growth or inhibits the nuclear translocation of pERK. Although GSK126 is a highly selective inhibitor of EZH2,^51^ our findings reflected the pharmacologic effects of EZH2 inhibition and might not necessarily recapitulate the intrinsic biological function of EZH2 itself. Notably, Adminasr et al reported that *EZH2* knockdown failed to reduce cellular proliferation.^52^ Future investigations should elucidate whether inhibition of pERK nuclear translocation also occurs with *EZH* knockdown and whether the efficacy of MEK inhibitor efficacy is enhanced under EZH2-deficient conditions. Second, regarding GSK126, the EZH2 inhibitor predominantly used in this study, is not approved for clinical use. Since the FDA-approved agent tazemetostat was employed in only a subset of experiments, further validation with tazemetostat would be necessary for facilitating clinical translation of our findings. Third, the in vivo evaluation of antitumor efficacy was constrained by the limited sample size (*n* = 5 per group). Future studies incorporating larger cohorts per treatment arm and/or in vivo validation across multiple cell lines might strengthen and extend these findings. Fourth, we were unable to elucidate the precise mechanism by which the EZH2 inhibitor could suppress the nuclear translocation of pERK. Although the EZH2-miR-30d-KPNB1 signaling pathway has been reported,^26^ we did not consistently observe decreased KPNB1 expression following EZH2 inhibitor treatment. Moreover, RNA sequencing revealed only minimal transcriptomic changes following EZH2 inhibitor treatment. RNA sequencing analyses of canonical EZH2/PRC2 target genes, consisting of 133 genes including HOXA genes, CDKN2A/B, FOXD3, and SOX11, and MAPK downstream transcriptional targets, consisting of 54 genese including FOS, JUN, EGR family genes, DUSP4/6, SPRAY2/4/, and CCND1, showed that GSK126 monotherapy induced only minimal transcriptional changes in both gene sets, with only one significantly differentially expressed gene identified in each analysis. In contrast, MAPK downstream transcriptional targets were markedly suppressed after selumetinib monotherapy and combination therapy. However, no significant differences in gene expression were detected between the combination therapy group and the selumetinib monotherapy group. Therefore, the observed inhibition of pERK nuclear translocation should be interpreted primarily as a finding derived from an immunostaining-based phenomenon that may be independent of canonical PRC2-mediated transcriptional regulation; we were unable to identify the specific molecular mediators involved. Thus, this observation should be regarded as hypothesis-generating, and further mechanistic investigation is warranted.

In summary, our findings indicate that the antitumor efficacy of an EZH2 inhibitor may be mediated through the inhibition of pERK nuclear translocation in NF1-associated MPNST. The present data show that a combination of an MEK inhibitor together with an EZH2 inhibitor could be an effective treatment for NF1-associated MPNST irrespective of PRC2 status.

## Supplementary Material

vdag221_Supplementary_Data

## Data Availability

The data generated in this study are within the article and its Supplementary Files. Data from RNA-seq analysis are publicly available in Gene Expression Omnibus.
